# Effect of various hepatectomy procedures on circulating tumor cells in postoperative patients: a case-matched comparative study

**DOI:** 10.3389/fmed.2023.1209403

**Published:** 2023-09-28

**Authors:** YongRong Lei, XiShu Wang, YiChen Tian, Rong Xu, Jun Pei, YuNa Fu, Heng Sun, YaNi Wang, Ping Zheng, Feng Xia, JianHua Wang

**Affiliations:** ^1^Key Laboratory of Biorheological Science and Technology (Ministry of Education), College of Bioengineering, Chongqing University, Chongqing, China; ^2^Key Laboratory of Hepatobiliary and Pancreatic Surgery, Institute of Hepatobiliary Surgery, Southwest Hospital, Third Military Medical University (Army Medical University), Chongqing, China

**Keywords:** circulating tumor cells, laparoscopic liver resection, open hepatectomy, recurrence, EMT, liquid biopsy, metastasis

## Abstract

**Background:**

The objective of this study is to elucidate the prevalence of systemic circulating tumor cells (CTCs) prior to and following resection of hepatocellular carcinoma (HCC), and to compare the disparities in postoperative CTCs in terms of quantity and classifications between the open liver resection (OPEN) and laparoscopic liver resection (LAP) cohorts.

**Patients, materials, and methods:**

From September 2015 to May 2022, 32 consecutive HCC patients who underwent laparoscopic liver resection at Southwest Hospital were retrospectively enrolled in this study. The clinicopathological data were retrieved from a prospectively collected computer database. Patients in the OPEN group matched at a 1:1 ratio with patients who underwent open liver resection during the study period on age, gender, tumor size, number of tumors, tumor location, hepatitis B surface antigen (HBsAg) positivity, alpha-fetoprotein (AFP) level, TNM and Child-Pugh staging from the database of patients to form the control group. The Can-Patrol CTC enrichment technique was used to enrich and classify CTCS based on epithelial-mesenchymal transformation phenotypes. The endpoint was disease-free survival (DFS), and the Kaplan–Meier method and multiple Cox proportional risk model were used to analyze the influence of clinicopathological factors such as total CTCs and CTC phenotype on prognosis.

**Results:**

The mean age of the 64 patients with primary liver cancer was 52.92 years (23–71), and 89.1% were male. The postoperative CTC clearance rate was more significant in the OPEN group. The total residual CTC and phenotypic CTC of the LAP group were significantly higher than those of the OPEN group (*p* = 0.017, 0.012, 0.049, and 0.030, respectively), which may increase the possibility of metastasis (*p* = 0.042). In Kaplan–Meier analysis, DFS was associated with several clinicopathological risk factors, including Barcelona Clinical Liver Cancer (BCLC) stage, tumor size, and vascular invasion. Of these analyses, BCLC Stage [*p* = 0.043, HR (95% CI) =2.03(1.022–4.034)], AFP [*p* = 0.007, HR (95% CI) =1.947 (1.238–3.062)], the number of positive CTCs [*p* = 0.004, HR (95% CI) =9.607 (2.085–44.269)] and vascular invasion [*p* = 0.046, HR (95% CI) =0.475 (0.22–1.023)] were significantly associated with DFS.

**Conclusion:**

In comparison to conventional OPEN technology, LAP technology has the capacity to augment the quantity of epithelial, mixed, and mesenchymal circulating tumor cells (CTCs). Following the surgical procedure, there was a notable increase in the total CTCs, epithelial CTCs, and mixed CTCs within the LAP group, indicating a potential drawback of LAP in facilitating the release of CTCs.

## 1. Introduction

HCC is one of the most common malignancies around the world (1). The treatment of HCC is developing rapidly and includes hepatectomy, targeted system treatment, new internal and external radiotherapy technologies and liver transplantation (2). Radical hepatectomy remains the primary treatment for primary hepatocellular carcinoma (HCC). While two surgical methods, laparoscopic hepatectomy (LAP) and open hepatectomy (OPEN), are available, the 5-year survival rate of HCC patients remains low due to the high early recurrence rate. Research has demonstrated that recurrence after tumor resection and shorter recurrence-free survival are closely associated with circulating tumor cells (CTCS) (3, 4). Consequently, assessing CTCS before and after radical hepatectomy is essential to improve the prognosis of HCC patients (5–8). Epithelial-mesenchymal transition (EMT) is a dynamic process that results in the complete or partial loss of epithelial cell characteristics and the acquisition of a mesenchymal phenotype, thereby enabling cancer cells to migrate to distant sites and increasing their risk of metastasis (9–11). Compared with the epithelial CTC, the mesenchymal phenotype is more aggressive, and surgical manipulation may facilitate the progression of the EMT (12, 13). Although the techniques for separating and characterizing CTC based on the physical properties of CTC or the expression of cell surface antigens have been reported (14, 15), the methods based on epithelial antigens may not be able to detect some aggressive CTC subgroups that may have experienced EMT (16). To address this issue, a recent publication has presented an optimized version of Can-Patrol^TM^ CTC enrichment based on RNA *in situ* hybridization (RNA-ISH). This technique enables the characterization and classification of CTCs using epithelial and mesenchymal markers (such as EpCAM, E-cadherin, CK8/18/19, vimentin, and Twist) with high collection efficiency, making it ideal for many types of cancer. Our study enriched and classified CTCs from patients with HCC using Can-Patrol™ CTC enrichment and RNA-ISH analysis, since few studies have examined EMT in CTCs from HCC patients. Furthermore, we examined the association between CTC subgroups and patient characteristics and outcomes. Additionally, we investigated the relationship between patient characteristics and outcomes and CTC subgroups (17–19). Several small-scale studies have been conducted to assess the effects of laparoscopic liver resection versus open liver resection on CTCs, however, the results are not consistent. As part of this study, we compared the positive rate of CTCs before and after hepatectomy and analyzed the effects of different surgical methods on postoperative CTCs. Furthermore, we evaluated the prognosis of patients with HCC after different surgical resection methods.

## 2. Materials and methods

### 2.1. Patient samples

**Figure 1:**
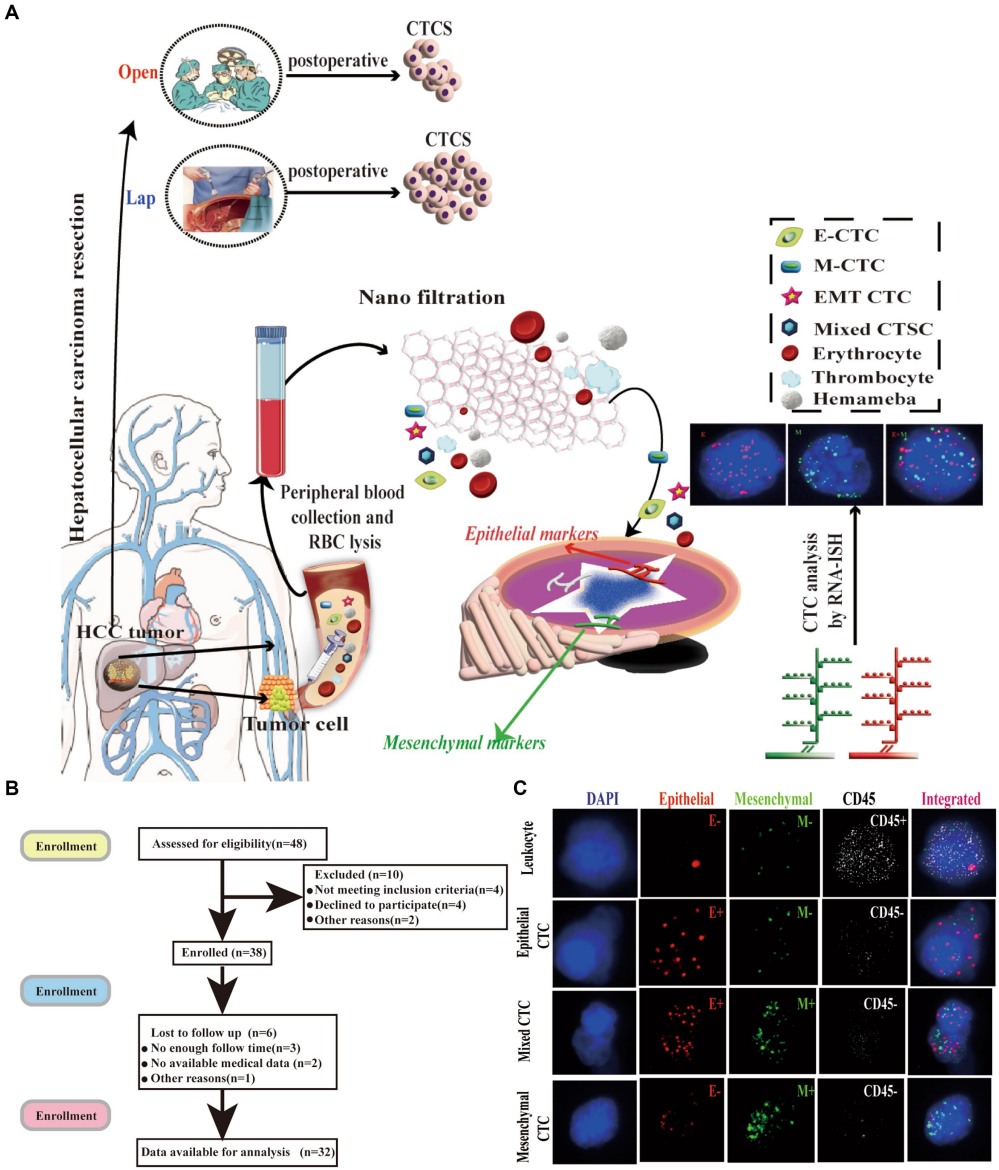
Isolation of CTCs and RNA-ISH analysis of blood samples from patients with HCC treated with laparoscopic hepatectomy and open hepatectomy. **(A)** Process of CTC isolation and detection by Can-Patrol™ CTC-enrichment and *in situ* hybridization (ISH). **(B)** Flow chart of patient enrolment. **(C)** CTCs were detected and classified using EMT markers. CD45 staining of leukocytes (white fluorescence). Epithelial (EpCAM and CK8/18/19, red fluorescence) and mesenchymal markers (vimentin and Twist, green fluorescence) were used on CTCs. Cells were analysed using a 100x objective.

From September 2015 to May 2022, 32 consecutive patients with hepatocellular carcinoma (HCC) who underwent laparoscopic liver resection at the Institute of Hepatobiliary Surgery, Southwest Hospital, Chongqing, China were enrolled in this study. The diagnosis of HCC was confirmed by histopathological examination of the operative specimens, and the clinicopathological data were retrieved from a prospectively collected computer database (Southwest Hospital Scientific Research Platform). Patients in the study group were matched at a 1:1 ratio with patients who underwent open liver cancer resection during the study period. Matching criteria included age, gender, tumor size, number of tumors, tumor location, hepatitis B surface antigen (HBsAg) positivity, alpha-fetoprotein (AFP) level, TNM and Child-Pugh staging, which were all obtained from the database of patients to form the control group. The same inclusion and exclusion criteria were applied to both the study and control groups (Figure 1B).

The study was approved by the Ethics Committee of the First Affiliated Hospital of Army Military Medical University, PLA (KY2017047). Written informed consent was obtained from the patients and their families.

All patients underwent the same preoperative evaluation protocol, which included abdominal ultrasonography, computed tomography (CT) scans of the chest and abdomen, CT angiography of the hepatic artery, hepatic vein, and portal vein, electrocardiography (ECG), and blood biochemistry. Additionally, epithelium-mesenchymal phenotype circulating tumor cells (CTCs) in the peripheral blood were detected in all patients 3 days before and post-surgery. Liver function tests were assessed using the Pugh-Child grading system and the indocyanine green clearance test at 15 min (ICG-R15).

### 2.2. Isolation of CTCs with the Can-Patrol™ system and tri-color RNA-ISH assay

To separate and detect CTCs, we used the Can-Patrol^TM^ system (20–22). We collected 15 mL samples of the peripheral blood of patients with HCC patients 3 days before and after the operation. Then, the samples were placed in Ethylene Diamine Tetraacetic Acid (EDTA)tubes and centrifuged. The cell beads were collected, and the supernatant was discarded. Subsequently, 5 mL of PBS was added to the tube, and the cell pellet was resuspended. The high-concentration cell suspension was then passed through the filter membrane under vacuum conditions, thus allowing the CTCs to be collected on the filter membrane (19, 23, 24).

We adopted multiple RNA-ISH technologies and designed three sets of nucleic acid probes to detect the expression of the epithelial-mesenchymal transition (EMT) marker, a leukocyte marker (CD45), epithelial markers (CK8, 18 and 19, and EpCAM), and mesenchymal markers (Twist and Vimentin) in circulating tumor cells (CTCs). We used a fluorescence optical microscope to quantitatively analyze the cells, with red used to mark epithelial cells, green used to mark mesenchymal cells, and white used to mark CD45^+^ cells. Additionally, 4, 6-diamidino-2-phenylindole (DAPI) was used to stain the cells’ nuclei (23, 25). The primer sequences used by the probes are detailed in Supplementary Table S2 (26) (Figure 1A; Supplementary Figure S9).

### 2.3. Surgical procedures

#### 2.3.1. LAP procedures

The patient was placed in the supine position, and the main and auxiliary operating holes were established in sequence. After freeing the liver, the first hepatic hilar occlusion band was preset, and the incision line was delineated according to preoperative imaging examination, intraoperative ultrasound, or the ischemic area after anatomical occlusion of the corresponding hepatic segment. The resection margin should be at least 2 cm from the tumor edge. The liver parenchyma was severed with an ultrasonic knife, and the tubes with a diameter greater than 3 mm were clamped with continuous titanium clips. The tubes with a smaller diameter were directly closed with the ultrasonic knife. The cross-section was stopped using an ultrasonic scalpel and bipolar coagulation, and the wound was then covered with hemostatic gauze. There was no active bleeding, the plasma drainage tube is placed under the liver section and led out from the abdominal wall. As soon as the pneumoperitoneum was removed, each puncture hole was sutured (27, 28). Special instruments used in this procedure included: trocar (CB12LT, Ethicon, Mexico), electric endoscopic arthroscopic linear cutting and stapler (PSE45A, Ethicon, Mexico), electric endoscopic linear cutting and stapler and staple cartridge (GCFRGW, Ethicon, Mexico), Ultrasonic High-Frequency Surgical Integrated System Ultrasonic Blade (HAR36, Ethicon, Mexico), Burst Clip Applier and Clips (ER320, Ethicon, Mexico), Disposable Central Venous Catheter Kit, (4161211P, bra, Germany).

#### 2.3.2. OPEN procedures

Both OPEN and LAP procedures were anesthetized using the same method. Under general anesthesia, the patients were placed in a “human”-shaped supine position. According to the specific location of the lesion, an incision was made at the midline of the abdomen or the right subcostal border, and the perihepatic ligaments were dissociated to fully reveal the location of the lesion. Ultrasonic scalpels were used to separate liver parenchyma, lesion tissue was removed, bleeding was actively controlled, and bile leaks were checked. Blood vessels and parenchymal tissues were dissected with the Cavitron ultrasonic surgical aspirator, and bleeding was stopped with bipolar coagulation (29, 30).

### 2.4. Postoperative care

Uniform care and monitoring were administered to all patients postoperatively. Blood tests and liver function tests were conducted on days 1, 3, and 7 subsequent to the surgical procedure. Removal of the abdominal drainage tube was contingent upon the presence of serous drainage fluid and absence of bile leakage. Additionally, ultrasound imaging was typically conducted prior to discharge.

### 2.5. Statistical methods

Correlation analysis was conducted using Pearson, Spearman, and Kendall’s tau-b correlations with the Statistical Package for Social Sciences (SPSS version 26, IBM Corp) (31). Categorical data and measurement data were evaluated using the *χ*^2^ test and *t*-test, respectively. Prognostic factors of early recurrence were assessed through univariate and multivariate Cox regression analyses. The association between recurrence and prognostic factors was determined using a Kaplan–Meier survival analysis, with log-rank tests employed to identify any differences in the curves. *p* value<0.05 was considered statistically significant.

## 3. Results

### 3.1. Patient characteristics

Among the patients included in the study, 64 patients with HCC included in the study, provided a total of 128 blood samples. The characteristics of the study participants are listed in Supplementary Table S1. There were 64 patients with HCC treated with resection (57 men and 7 women), aged 23–71 years on average. Among the reported cases of HCC, nine were well-differentiated (14.1%), 50 (78.1%) were moderately differentiated, and five (7.2%) were poorly differentiated. The BCLC stage A patient count was 38 (59.5%) and the BCLC stage B patient count was 26 (40.6%). The TNM staging analysis revealed 44 (68.8%) patients with TNM stages I-II and 20 (31.3%) patients with TNM stages III. The majority of patients in this cohort (63 cases, 98.4%) had cirrhosis of the liver, of whom 33 (51.6%) had intrahepatic metastasis and 34 (53.1%) had tumor vascular invasion (Supplementary Table S1).

Baseline clinical features and tumor features were shown in Table 1; Supplementary Table S3. No significant differences were found between the two groups in terms of the operation time, blood loss, and a number of patients not requiring transfusions. Kringle’s median maneuver time was similar between the two groups, about 1 hour. The baseline data did not differ significantly between the groups (*p* > 0.05).

**Table 1:** Baseline characteristics of patients during treatment.

Clinical characteristics	Laparoscopic	Open	*p* ^#^
No. of patients	32	32	–
Gender (Male/Female)	30/2	27/5	0.426
Age (≤60/>60 years)	22/10	25/7	0.572
Tumor Number (1/>1)	30/2	26/6	0.257
Differentiation (H/M/L)	4/26/2	4/25/3	1.000
BCLC Stage(A/B)	23/9	18/14	0.193
Tumor Size (≤5/>5 cm)	25/7	18/14	0.062
AFP (<20/20–400/>400 μg/L)	10/15/7	14/9/8	0.330
TNM (I-II/III)	26/6	20/12	0.095
MVI(Yes/No)	17/15	17/15	1.000
HBsAg (+/−)	25/7	30/2	0.148
Cirrhosis (Yes/No)	29/3	31/1	0.613
ICG (<4.7/>4.7%)	13/19	20/12	0.133
Child-Pugh classification(A/B)	29/3	30/2	1.000
WBC (<9.5/>9.5 × 10^9^/L)	30/2	31/1	0.554
RBC (<4/>4 × 10^9^/L)	2/30	2/30	1.000
Direct Bilirubin (≤4/>4 μmol/L)	15/17	17/15	0.617
Total Bilirubin (<20/>20 μmol/L)	23/9	24/8	0.777
ALT (<40/>40 IU/L)	19/13	20/12	0.798
Edmondson stage (H/M/L)	4/26/2	5/23/4	0.696
APRI (<2/>2)	27/5	29/3	0.354
Neutrophil (≤4/>4)	20/12	20/12	1.000
PT (<13/>13Sec)	30/2	30/2	1.000
PLT (≤100/>100 × 10^9^/L)	11/21	5/27	0.083
AST (<40/>40 IU/L)	21/11	18/14	0.442
ALB (≤35/>35 g/L)	1/31	2/30	0.500
Tumor location (R/L/R + L)	18/12/2	21/6/5	0.184

^*^Value expressed in the median with a range in parentheses. ^#^Laparoscopic hepatectomy group compared with open hepatectomy group.

### 3.2. EMT is a key step in CTCs formation

The number, type, and expression of specific genes and proteins in CTCs are closely related to tumor progression (32). For the separation, classification and identification of CTCs, Can-Patrol’s second-generation typing detection technology was used (Figure 1A). After separating the CTCs and extracting 10 mL of peripheral blood from two groups of patients who underwent different surgical methods, the red blood cells (RBCs) in the peripheral blood were lysed and then filtered through a nanomembrane. To enrich CTCs, tumor cells were separated from white blood cells (WBCs) based on their sizes. In the second step of identification, a new type of multiple mRNAs *in situ* analysis (MRIA) method was used to locate the enriched CTCs with a specific nucleic acid to achieve the purpose of typing and identifying CTCs. Red and green fluorescent signals were used to represent epithelial and mesenchymal gene expression, respectively. The white fluorescent signal represents CD45 gene expression (WBC marker, Figure 1C; Supplementary Table S2).

To classify and count CTCs, we used RNA-ISH analysis to divide CTCs into the following three subgroups: (1) epithelial CTC(E > M-CTC), (2) epithelial/mesenchymal mixed CTC (E ≈ M-CTC), (3) mesenchymal CTC (M > E-CTC) (Figure 1B; Supplementary Figure S7). Patient demographics were shown in Supplementary Table S1. Most of the 144 patients (90%) had CTC positive (EpCAM^+^CK^+^ CD45^−^DAPI^+^) in their blood samples, and 16 patients (10%) had CTC negative (EpCAM^−^CK^+^CD45^−^DAPI^+^). Our study found that among CTC-positive patients with HCC, 10 cases (6.3%) were epithelial (type E), 21 cases (75.6%) were mesenchymal (type M), and 13 cases (8.1%) were mixed (mixed type) (Supplementary Table S3).

### 3.3. Differences in total CTCs and phenotypes of CTCs in blood samples from patients with HCC before and after the two surgical regimens

**Figure 2:**
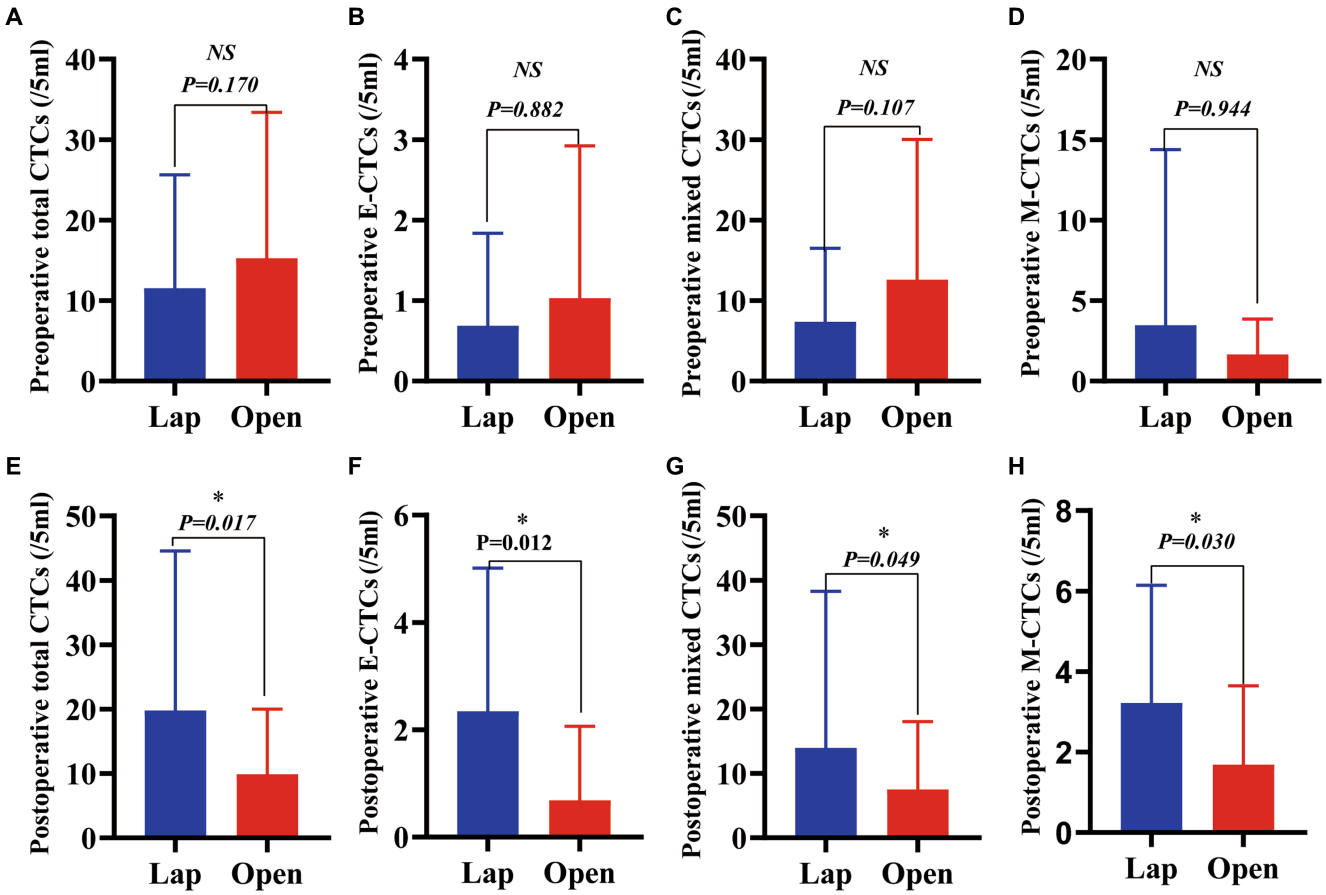
A comparison of total CTCs and CTC phenotypes in blood samples from patients with HCC before and after these two surgical regimens. **(A)** Preoperative total CTCs (/5 mL) (*p* = 0.170); **(B)** Preoperative epithelial CTCs (/5 mL) (*p* = 0.882); **(C)** Preoperative mixed CTCs (/5 mL) (*p* = 0.107); **(D)** Preoperative mesenchymal CTCs (/5 mL) (*p* = 0.944); **(E)** Postoperative total (/5 mL) (*p* = 0.017); **(F)** Postoperative epithelial CTCs (/5 mL) (*p* = 0.012); **(G)** Postoperative mixed CTCs (/5 mL) (*p* = 0.049); **(H)** Postoperative mesenchymal CTCs (/5 mL) (*p* = 0.030).(Value expressed in median with range in parentheses).

We collected the blood of patients 3 days before surgery and 3 days after surgery for CTC detection and analysis, and the results showed that 96.88% of patients were CTC positive, including 93.75% in the LAP group and 100% in the OPEN group. Furthermore, there were no significant differences in preoperative total CTCs, epithelial CTCs, mixed CTCs, and mesenchymal CTCs between the two groups (*p* = 0.170, 0.882, 0.107, 0.944, Figures 2A–D).

The changes in the number of total CTCs before and after operation between the two groups are different. The number of cases increasing the total CTCs in the LAP and the OPEN groups were 21 and 10, respectively. There was a significant difference between the two groups (*p* = 0.006) Further sorting of CTC phenotypes revealed that the LAP group had significantly higher levels of total CTCs, epithelial CTCs, mixed CTCs and mesenchymal CTCs compared to the OPEN group (*p* values are 0.017, 0.012, 0.049 and 0.030, respectively) (Figures 2E–H; Table 2).

**Table 2:** Comparison between open liver resection and laparoscopic liver resection, and observation of changes in preoperative and postoperative circulating tumor cell count.

Variable	Laparoscopic (CTCs/mL)	Open (CTCs/mL)	*p* ^#^
Preoperative total CTCs^*^	7.50 (0.00–65.00)	10.00 (1.00–97.00)	0.17
Postoperative total CTCs^*^	15.5 (0.00–118.00)	7.00 (0.00–46.00)	0.017
Preoperative epithelial CTCs^*^	0.00 (0.00–4.00)	0.00 (0.00–7.00)	0.882
Postoperative epithelial CTCs^*^	2.00 (0.00–8.00)	0.00 (0.00–6.00)	0.012
Preoperative mixed CTCs^*^	3.00 (0.00–38.00)	7.00 (0.00–94.00)	0.107
Postoperative mixed CTCs^*^	9.00 (0.00–115.00)	4.00 (0.00–46.00)	0.049
Preoperative mesenchymal CTCs^*^	1.00 (0.00–62.00)	1.00 (0.00–8.00)	0.944
Postoperative mesenchymal CTCs^*^	3.00 (0.00–10.00)	1.00 (0.00–7.00)	0.03
The total amount of CTC increased after surgery	21	10	0.006
Preoperative Total CTCs positive rate	30/32	32/32	0.492
Postoperative Total CTCs positive rate	31/32	29/32	0.613

^*^Value expressed in the median with a range in parentheses. ^#^Comparing laparoscopic hepatectomy with open hepatectomy.

**Figure 3:**
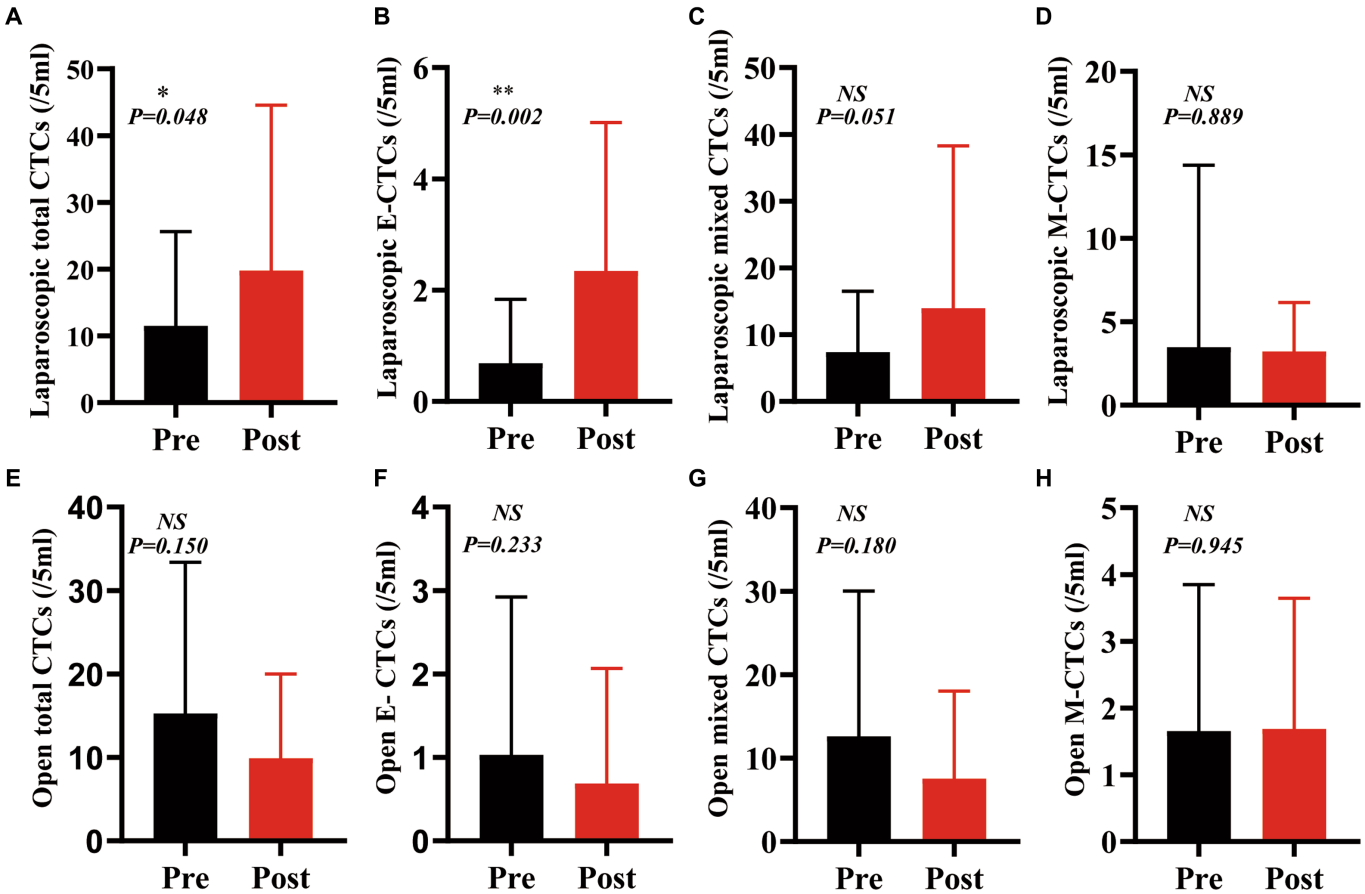
The counts of CTCs and phenotypes after laparoscopic hepatectomy were significantly higher than those before the operation, but there was no significant change after open hepatectomy. **(A)**: Total CTCs increased after laparoscopic hepatectomy (*p* = 0.048); **(B)** Epithelial CTCs increased after laparoscopic hepatectomy (*p* = 0.002);**(C,D)** Mixed CTCs and mesenchymal CTCs did not change after laparoscopic hepatectomy (*p* = 0.051,0.889); **(E–H)** Total CTCs, epithelial CTCs, mixed CTCs, and mesenchymal CTCs did not change significantly after open hepatectomy (*p* = 0.151, 0.233,0.180, and 0.945) (Paired *t*-test, GraphPad prism).

The number of total circulating tumor cells (CTCs) was significantly increased after laparoscopic hepatectomy (*p* = 0.048, Figure 3A). Moreover, the number of epithelial CTCs after laparoscopic hepatectomy was significantly higher than that before the operation (*p* = 0.002, Figure 3B). However, there was no significant difference between the number of mixed CTCs and mesenchymal CTCs before and after the operation (*p* = 0.051, 0.889, Figures 3C,D). Additionally, the preoperative and postoperative differences of total CTCs, epithelial CTCs, mixed CTCs, and mesenchymal CTCs in the OPEN group were not significant (*p* values were 0.150, 0.233, 0.180, and 0.945, respectively; see Figures 3E–H).

### 3.4. The changes in clinical indicators after the two operations were similar

**Figure 4:**
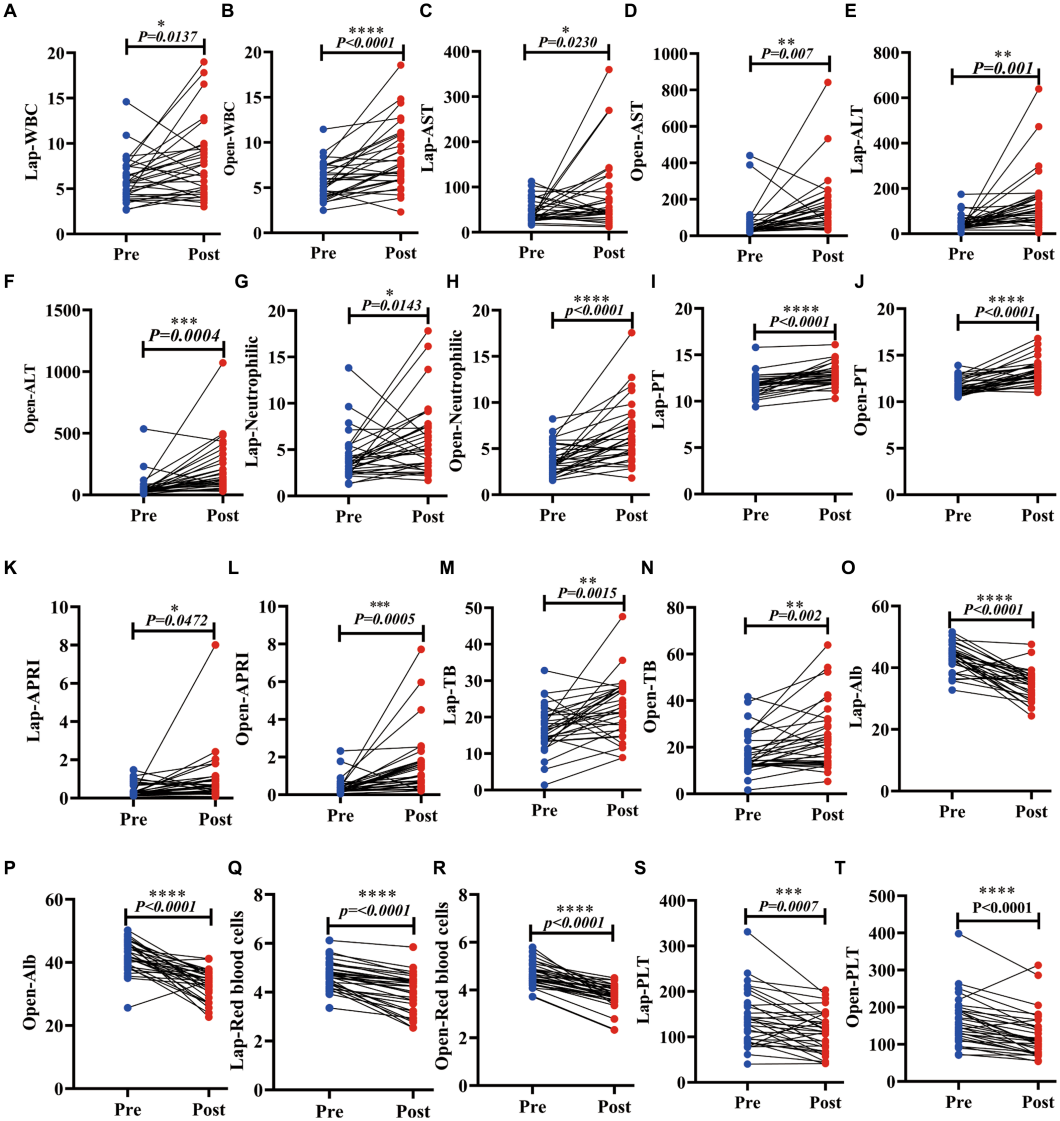
Laparoscopic hepatectomy and open hepatectomy significantly changed various clinical indicators of liver function in patients with liver cancer. **(A–N)**: The LAP and the OPEN. The WBC, AST, ALT, neutrophil, PT, APRI, and TB were increased (*p* < 0.05); **(O–T)**: Postoperative ALB, PLT and red blood corpuscle of laparoscopic hepatectomy and open hepatectomy decreased (*p* < 0.05).

To explore the difference between two different surgical methods in the recovery of patients’ liver function after surgery, we collected data on liver function indexes 3 days before and within 1 week after surgery. The study found that postoperative WBCs, aspartate aminotransferase (AST), alanine aminotransferase (ALT), neutrophils, AST to platelet count ratio index (APRI), total bilirubin (TBil), and prothrombin time (PT) increased in the laparoscopic (LAP) group (*p* = 0.0137, 0.0230, 0.0010, 0.0143, <0.0001, 0.0472 and 0.0015), while platelets (PLTs), albumin (ALB), and the number of red blood cells (RBCs) decreased after surgery (*p* = 0.0007, <0.0001, <0.0001). Similarly, in the open group, WBC, AST, ALT, neutrophils, APRI, TBil, and PT also increased (*p* = <0.0001, 0.0070, 0.0040, <0.0001, 0.0020, <0.0001). PLTs, ALB, and the number of RBCs also decreased after surgery (*p* < 0.0001). In the two radical operations for liver cancer within 1 week after surgery, the liver showed increased inflammation indicators due to damage, the clotting time became longer, and PLTs and ALB decreased due to blood loss. There was almost no change after AFP. They have similar trends in both OPEN and LAP surgical methods (Figure 4; Supplementary Figure S1; Supplementary Table S4); thus, the surgical methods harm liver function in the short term after surgery, and laparoscopy does not reduce the harm.

### 3.5. The prognostic effects of LAP and OPEN were different

**Figure 5:**
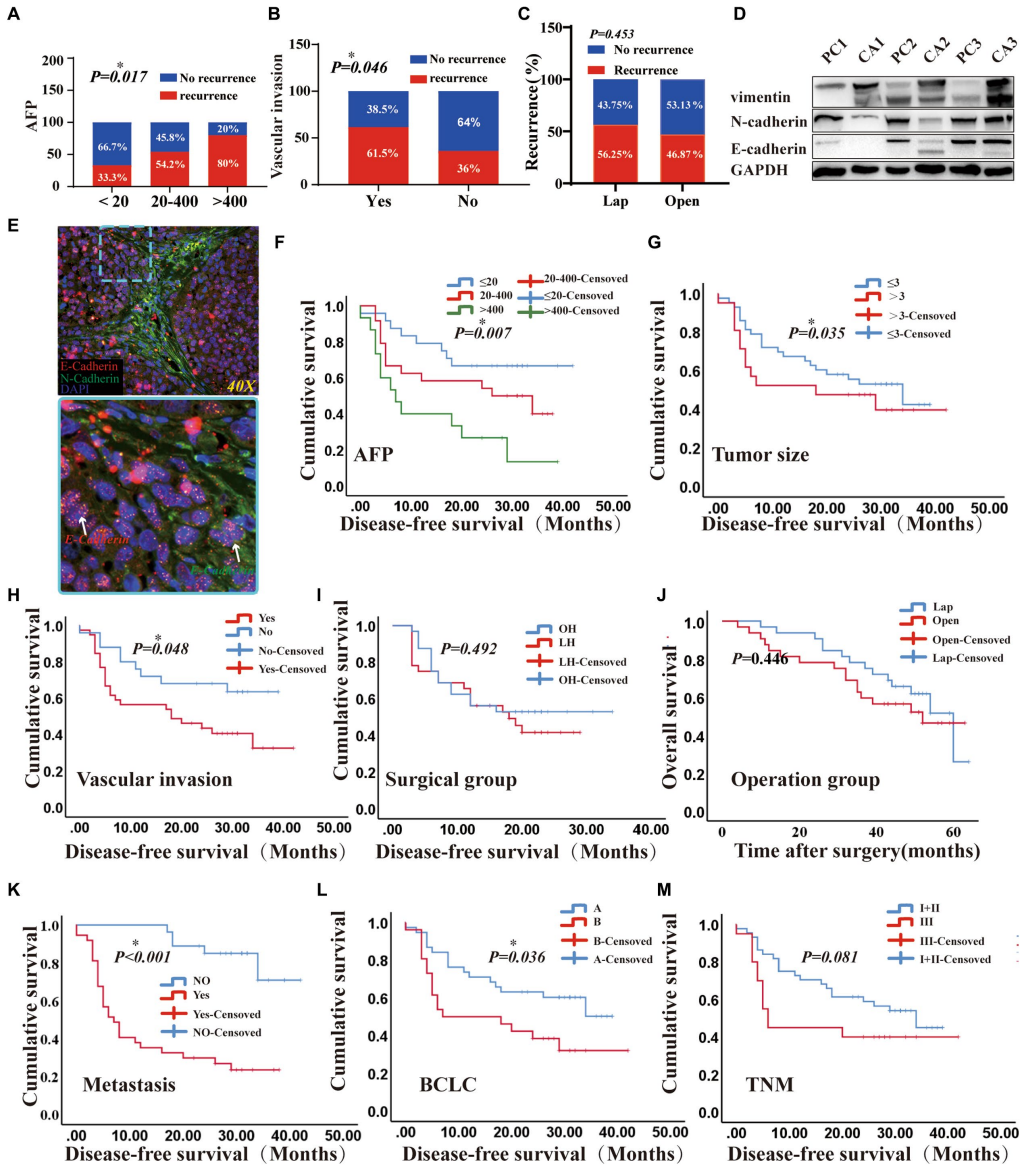
The prognostic effects of laparoscopic hepatectomy and open hepatectomy were different. **(A)** A positive correlation was found between AFP classification and relapse (*p* < 0.05); **(B)** A positive correlation was found between vascular invasion and recurrence (*p* < 0.05); **(C)** Comparison of Laparoscopic and Open Surgery in terms of Short-term Recurrence (*p* > 0.05). **(D)** Among the three pairs of cancer and adjacent liver cancer tissues, the epithelial marker (E-Cadherin) in cancer tissue was higher than that in adjacent cancer tissue, and the mesenchymal marker (vimentin, N-Cadherin) in cancer tissue was lower than that in adjacent cancer tissue; **(E)** Cryosections were double-stained with N-cadherin and E-cadherin -specific antibody. (5-μm frozen sections, original magnifications, ×40). **(F–I)**: Kaplan–Meier methods: AFP, Tumour size, Vascular invasion, surgical methods result (*p* were 0.007, 0.035, 0.048, 0.492). **(J)** overall survival (OS) of the patients with two different surgical groups (Log-rank test *p*-value = 0.446). **(K–M)**: Kaplan–Meier methods: metastasis, BCLC, TNM staging results (<0.001, 0.036, 0.081). (DFS and OS results by Cox regression analysis).

There was no significant difference in the number of total CTCs, epithelial CTCs, mixed CTCs, and mesenchymal CTCs between the two groups before hepatectomy. However, the clearance rate of CTCs was more evident in the OPEN group. Postoperative residual total CTCs and various phenotypes CTCs in the LAP group were significantly higher than those in the OPEN group (*p* values were 0.017, 0.012, 0.049 and 0.030, respectively; Figures 2E–H). Additionally, the intrahepatic metastasis rate in the LAP group was higher than that in the OPEN group (*p* = 0.042; Supplementary Figure S3). During the follow-up period, 33 cases (51.6%) of all patients had a recurrence of liver cancer, with 15 cases in the OPEN group and 18 cases in the LAP group. All cases were related to the recurrence of extrahepatic liver cancer, and AFP was positively correlated with recurrence. Eighty percent of patients with an alpha-fetoprotein (AFP) level higher than 400 had recurrence (*p* = 0.017; Figure 5A). Additionally, the recurrence rate was higher in patients with vascular invasion (*p* = 0.046; Figure 5B). The median follow-up time for the entire population was 41.3 months (range 6–193.5), with a 1-year and 2-year disease-free survival (DFS) rate of 37.5 and 14.1%, respectively (Supplementary Figure S2A). The recurrence rate was similar between the laparoscopic (LAP) and open (OPEN) groups, with 18 (56.3%) and 15 (46.9%) cases, respectively (*p* = 0.453; Figure 5C; Supplementary Figure S2B). Of the 33 recurrences, three and two occurred in the lungs of the LAP and OPEN groups, respectively (*p* = 0.16). Of the recurrent cases, 10 received radiofrequency treatment after surgery, two received PDL-1 treatment and two underwent interventional treatment. Kaplan–Meier analysis showed that DFS was associated with several clinicopathological risk factors, including BCLC stage, tumor size, and vascular invasion (all *p* < 0.05, Figure 5).

The prognostic effects of laparoscopic (LAP) and open (OPEN) surgery were different (Figure 5). *In vivo* experiments utilizing BALB/c nude mice were conducted to demonstrate that the tumors derived from highly metastatic human liver cancer cells, designated 97H, exhibit the EMT signature (Figure 5E). Microvascular infiltration and metastasis were usually found at the time of liver resection and were significantly associated with a poor prognosis (33). Our results showed that patients with vascular invasion had a poorer survival than those without (Yes vs. not reached, median: 27.7 vs. 28.59, Log Rank *p* = 0.048) (Figures 5B,H). Additionally, the estimated median survival time of patients in the Barcelona Clinic Liver Cancer (BCLC) (A) and BCLC (B) groups was 26.83 months and 19.78 months, respectively, and the survival curves of the patients were significantly different (Log Rank *p* = 0.036), with BCLC (A) being better than BCLC (B) (Figure 5L). Additionally, patients with larger tumors (>3 vs. <3, median: 21.53 vs. 28.87, Log Rank *p* = 0.035) and intrahepatic metastasis (Yes vs. Not Reached, median: 14.85 vs. 37.47, Log Rank *p* < 0.0001) had a worse prognosis (Figures 5G,K). Furthermore, patients with AFP levels >400 μg/L had a worse prognosis than those with <20 μg/L and 20-400 μg/L (median: 14.07vs 31.38, 22.89, Log Rank *p* = 0.007, Figure 5F). However, after two surgeries, the survival curves of patients were similar (*p* = 0.492, Figure 5I). Moreover, the operation time, TNM stage, ALB and blood loss were not associated with prognosis in the short-term follow-up results (*p* = 0.492, 0.081, 0.472 vs. 0.89; Figure 5; Supplementary Figures S3, S6). Kaplan Meier analysis of DFS and overall survival (OS) were conducted for patients with different circulating tumor cell (CTC) phenotypes. The results showed that there were no statistically significant differences in DFS and OS among the groups of totals, epithelial, mixed and mesenchymal CTCs (*p* > 0.05; Supplementary Figure S8). Additionally, operation time, TNM stage, ALB, and blood loss were not associated with the prognosis during short-term follow-up. (*p* = 0.446; Figure 5J).

The primary objective of this study was to investigate the prognostic factors associated with disease-free survival (DFS) in patients classified as Child-Pugh stage A. To achieve this aim, thorough univariate and multivariate Cox analyses were performed. The comprehensive results are presented in Table 3, providing valuable insights into the relationships between various factors and DFS. The univariate analysis revealed significant associations between DFS and specific variables, including BCLC Stage [*p* = 0.043, HR (95% CI) = 2.03 (1.022–4.034)], AFP [*p* = 0.007, HR (95% CI) = 1.947 (1.238–3.062)], the number of positive CTCs [*p* = 0.004, HR (95% CI) = 9.607 (2.085–44.269)], and vascular invasion [*p* = 0.046, HR (95% CI) = 0.475 (0.22–1.023)]. To further investigate the significant factors identified in the univariate analysis, a subsequent multivariate analysis was conducted. Within the multivariate analysis, only the number of positive CTCs [*p* = 0.003, HR (95% CI) = 12.814 (2.331–70.432)] and AFP levels (>400, 20–400, <400) [*p* = 0.047, HR (95% CI) = 12.814 (2.331–70.432)] demonstrated meaningful associations with DFS. Notably, when comparing the two surgical methods employed (open group and LAP group), both the univariate and multivariate Cox analyses failed to identify any significant differences [*p* = 0.676, HR (95% CI) = 0.864 (0.435–1.715)].

**Table 3:** Multivariate and univariate Cox regression analysis of preoperative patient characteristics associated with HCC recurrence (*n* = 64).

Variables	Univariate analysis		Multivariate analysis	
	HR (95% CI)	*p*-value	HR (95% CI)	*p*-value
Gender (Male/Female)	0.767 (0.27–2.189)	0.623	NA	NA
Age (≤60/>60 years)	1.456 (0.588–3.457)	0.433	NA	NA
Tumor Size (≤5 />5 cm)	1.394 (0.685–2.838)	0.36	NA	NA
Tumor Number (≤1/>1)	0.978 (0.458–2.087)	0.953	NA	NA
Differentiation (H/M/L)	0.851 (0.447–2.087)	0.625	NA	NA
BCLC Stage (A/B)	2.030 (1.022–4.034)	**0.043**	1.524 (0.675–3.445)	0.311
AFP (>400,20–400, <400)	1.947 (1.238–3.062)	**0.007**	12.814(2.331–70.432)	**0.047**
CTC count (/mL)	1.007 (0.983–1.021)	0.851	NA	NA
WBC (≤9.5/>9.5/L)	1.244 (0.513–3.015)	0.629	NA	NA
E CTC (+/−)	1.024 (0.503–2.085)	0.948	NA	NA
Mixed CTC (+/−)	0.591 (0.243–1.433)	0.244	NA	NA
M CTC (+/−)	1.250 (0.594–2.63)	0.557	NA	NA
CTC (+/−)	9.607 (2.085–44.269)	**0.004**	12.814(2.331–70.432)	**0.003**
CTC (≤8.75/>8.75)	1.314 (0.659–2.622)	0.438	NA	NA
operation time (<242/>242)	1.211 (0.611–2.398)	0.584	NA	NA
surgical method (LAP/OPEN)	0.864 (0.435–1.715)	0.676	NA	NA
vascular invasion (+/−)	0.475 (0.22–1.023)	**0.046**	0.554 (0.242–1.269)	0.162
MVI (Yes/NO)	0.680 (0.338–1.368)	0.279	NA	NA
MVD (Yes/NO)	0.907 (0.409–2.015)	0.811	NA	NA
Blood loss (>300/<300)	1.047 (0.506–2.167)	0.101	NA	NA
Direct Bilirubin (<7/>7)	0.925 (0.355–2.411)	0.874	NA	NA
Total bilirubin (<20/>20)	0.241 (0.056–1.036)	0.056	NA	NA
ALT (<40/>40 IU/L)	0.708 (0.356–1.408)	0.325	NA	NA
TNM (I-II/III)	1.685 (0.826–3.439)	0.152	NA	NA
Edmondson (I-II/III-IV)	0.968 (0.295–3.177)	0.957	NA	NA
Postoperative increase in CTC	1.177(0.594–2.332)	0.64	NA	NA
Postoperative increase in ECTC	1.707(0.848–3.487)	0.134	NA	NA
Postoperative increase in Mix CTC	1.163(0.587–2.302)	0.665	NA	NA
Postoperative increase in M-CTC	0.627 (0.308–1.277)	0.199	NA	NA
APRI (≤1/>1)	1.068 (0.507–2.251)	0.863	NA	NA
Portal vein block time (<37/>37)	1.268 (0.638–2.519)	0.498	NA	NA
Neutrophil (<4/>4)	1.057 (0.525–2.127)	0.877	NA	NA
PT (<13/>13)	0.900 (0.215–3.766)	0.885	NA	NA
PLT (≤100/>100)	0.797 (0.359–1.772)	0.579	NA	NA
AST (≤45/>45)	1.838 (0.917–3.68)	0.089	NA	NA
ALB (≤35/>35)	1.668 (0.398–6.995)	0.484	NA	NA
ICG (≤10/>10%)	0.947 (0.477–1.879)	0.877	NA	NA

HR, hazard ratio; CI, confidence intervals; Bold font is used to describe *p* < 0.05, which is statistically significant.

## 4. Discussion

Currently, liver cancer resection is recognized as an effective treatment. The laparoscopic procedure has a history of more than 100 years, dating back to 1901 (34, 35). With the development of technology, at present, the LAP has been applied to tumors in the entire liver are, and has become one of the standard treatments for resection of the right anterior and left lateral lobes. A large number of studies have confirmed the safety and feasibility of LAP (36, 37). Compared with OPEN, LAP has the advantages of aesthetics, less blood transfusion, less tissue damage, less patient pain, and shorter hospital stay. Although the application of LAP has developed fast, the prognosis is still poor due to the high incidence of recurrence and metastasis (38). In recent years, the application of laparoscopic technology in gynecological tumors has caused some questionable problems. A multicenter, randomized, open-label, phase 3, non-inferiority trial (LACC) demonstrated that, in patients with early-stage cervical cancer, the risk of death after minimally invasive surgery is higher than that after a radical hysterectomy performed with a traditional open incision (laparotomy). The minimally invasive procedure is also associated with higher recurrence rates than open surgery (39). The authors of the LACC trial proposed that the poorer oncologic outcomes in patients undergoing laparoscopic radical hysterectomy may be due to the contamination of the pelvis at the time of colpotomy, as well as the potential for the flow of CO2 to spread cancer cells into the abdominal cavity. Animal experiments have also suggested that CO2 pneumoperitoneum can lead to heavier intraperitoneal and incisional metastasis than the open controls. Ost etc. demonstrated that CO2 pneumoperitoneum inhibited the peritoneal macrophage TNF-α secretion. And the inhibition of peritoneal macrophage TNF-α secretion may contribute to the development of port-site metastasis (40). In another study on a murine neuroblastoma, CO2 pneumoperitoneum increased intrahepatic metastasis too (41).

After surgery, the LAP group had more epithelial, mixed, and mesenchymal CTCs than the OPEN group. The reasons for this may be that pneumoperitoneum, which is a requirement for laparoscopic liver resection and is usually achieved with carbon dioxide, and the need to maintain intra-abdominal pressure during the procedure, may promote the entry of tumor cells into the blood circulation or abdominal cavity postoperatively. This effect is not seen with open liver resection. After open liver resection, CO2 pneumoperitoneum (CDP) had no effect on circulating tumor cells. The increased abdominal pressure caused by CDP in a short period of time may affect hemodynamics, hemoperfusion and the functioning of important abdominal organs such as the liver. Furthermore, LAP may induce EMT. In recent years, CTC has been regarded as a major source of recurrence and metastasis after liver resection (42) and has become a key point of entry for studying the process and mechanism of tumor metastasis and recurrence (43). The preoperative numbers of epithelial, mixed, and mesenchymal CTCs were similar in the LAP and OPEN groups. However, the marked increase in various phenotypic CTCs in the postoperative LAP group may be associated with EMT. Laparoscopic surgery has been found to reduce postoperative CTC in a small number of studies compared to open surgery, but the number of cases in these studies is relatively small, and there are no results of related prospective multi-center studies (44). After the operation, the total CTCs in the LAP group were significantly higher than those in the OPEN group. This may be the result of the intra-abdominal pressure which must be maintained during laparoscopic surgery, and the flow of CO2 which may promote tumor cells to enter the blood circulation. Additionally, the total CTCs, epithelial CTCs, and mesenchymal CTCs in the laparoscopic group were significantly increased compared to before the operation. To elucidate the importance of CTCs in HCC patients for EMT, we employed an advanced Can-Patrol CTC enrichment technology and hybridization technology. The CTCs were enriched and classified from blood samples. Patients with CTC-positive HCC have a higher risk of recurrence and a shorter recurrence-free survival period (45). Through phenotypic changes, CTCs penetrate blood vessels, including EMT, and spread from primary tumors (13, 46). Tumor progression is associated with changes in the ratio of epithelial to mesenchymal CTC (47).

CTCs are metastatic culprits and represent an attractive and promising alternative to tumor biopsy for management and genomic analysis (12, 48–50). Postoperative total CTCs, epithelial CTCs, and mixed CTCs in the LAP group increased significantly compared to preoperative levels. This may be due to the same factors mentioned above. There was no significant difference in DFS between the two groups, which may be attributed to the short follow-up period. Single-cell analysis has revealed that CTCs often contain heterogeneous cell populations, which may reflect the degree of intratumorally heterogeneity and have important implications for prognosis and treatment resistance (51, 52). Furthermore, CTCs often appear heterogeneous in their epithelial-mesenchymal transition (EMT) status (47, 53). The expression of the epithelial marker (E-Cadherin) was found to be higher in cancer tissue than in adjacent cancer tissue, while the expression of the mesenchymal marker (vimentin, N-Cadherin) was lower in cancer tissue than in adjacent cancer tissue. Additionally, it was observed that the presence of more circulating tumor cells with positive qualitative markers (vimentin, N-cadherin) was associated with a poorer prognosis for patients.

This study has several limitations. First, it was designed retrospectively with a relatively small sample size, resulting in an unbalanced queue in terms of CTC count and subset state. We will conduct large cohort studies in future investigations. Second, the Can-Patrol system used to collect CTCs in this study allowed particles less than 8 mm in diameter to pass through the filter without being detected. The detection efficiency of CTCs can be improved by using multiple detection techniques in future research. Finally, due to the time and compliance of the patients, we cannot detect the CTC count 7 days or 2 weeks after surgery. Future research will improve the related work.

## 5. Conclusion

In summary, our findings indicate that laparoscopic resection of hepatocellular carcinoma (HCC) leads to an increase in the quantities of epithelial, mixed, and mesenchymal circulating tumor cells (CTCs) compared to open resection (OPEN). Additionally, postoperative total CTCs, epithelial CTCs, and mixed CTCs in the LAP group exhibited a significant increase compared to preoperative levels. These results suggest that laparoscopic resection may potentially promote the release of CTCs and facilitate tumor metastasis in HCC patients. However, further investigations are required to elucidate the specific mechanisms underlying this phenomenon.

## Data availability statement

The original contributions presented in the study are included in the article/Supplementary material, further inquiries can be directed to the corresponding authors.

## Ethics statement

The study was conducted in accordance with the Declaration of Helsinki, and approved by the Ethics Committee of the First Affiliated Hospital of the Army Military Medical University ((B)2021012). All patients in the study signed and provided written informed consent.

## Author contributions

YL, XW, and FX: conception or design. FX and JP: provision of study materials or patients. YL and XW: collection and/or assembly of data. YL, XW, YW, YF, and HS: data analysis and interpretation. RX: Funding acquisition and Resources. YL, XW, FX, JW, and PZ: manuscript writing. YL, XW, YT, RX, JP, YF, HS, YW, PZ, FX, and JW: financial approval of manuscript. All authors contributed to the article and approved the submitted version.

## Abbreviations

BCLC stage: Barcelona Clinical Liver Cancer
AFP: Alpha-fetoprotein
WBC: White blood cell
ALB: Albumin
RBC: Red Blood Cell
ICG: Indocyanine green.
PLT: Platelets
PT: Prothrombin time
ICG: Indocyanine green
AST: Glutamic oxaloacetic transaminase
MVI: Microvascular tumor thrombus
APRI: Aspartate aminotransferase-platelet ratio
MVD: Micro vessel deity
ALT: Alanine aminotransferase
TNM: Tumor Node Metastasis
