# Supplementary material for: Effect of various hepatectomy procedures on circulating tumor cells in postoperative patients: a case-matched comparative study
Source: Front Med (Lausanne). 2023 Sep 28;10:1209403. doi: 10.3389/fmed.2023.1209403 (PMC10568028; doi:10.3389/fmed.2023.1209403)
Supplement: Supplementary file 1 [file Data_Sheet_1.docx]

Supplementary Material

# Supplementary Tables

## Supplemental Table 1: Difference of circulating tumor cells in both resection surgery preoperative assessment of diagnosis of hepatocellular carcinoma

| Variable | N | % |
| --- | --- | --- |
| Gender (Male/Female) | 57/7 | 89.1/10.9 |
| Age (≤60/>60 years) | 49/15 | 76.6/23.4 |
| Tumor Size(≤5/>5cm) | 43/21 | 67.2/32.8 |
| Tumor Size (≤3/>3cm) | 22/42 | 34.4/65.6 |
| Tumor Number (≤1/>1) | 56/8 | 87.5/12.5 |
| Differentiation(H/M/L) | 9/50/5 | 14.1/78.1/7.8 |
| BCLC Stage(A/B+C) | 38/26 | 59.4/40.6 |
| Metastasis (Yes/No) | 33/31 | 51.6/48.4 |
| AFP (≤20/20-400/>400μg/L) | 24/25/15 | 37.5/39.1/23.4 |
| Operation time (≤239/>239ml) | 32/32 | 50/50 |
| CTC (+/-) | 62/2 | 96.9/3.1 |
| E CTC (+/-) | 23/41 | 35.9/64.1 |
| Mixed CTC (+/-) | 54/10 | 84.4/15.6 |
| M CTC (+/-) | 41/23 | 64.1/35.9 |
| Pringle Time (≤37/>37) | 32/32 | 50/50 |
| WBC (≤9.5/>9.5) | 12/52 | 18.8/81.3 |
| RBC (≤4/>4) | 60/4 | 93.8/6.3 |
| Vascular Invasion (+/-) | 34/30 | 53.1/46.9 |
| MVI(Yes/No) | 34/30 | 53.1/46.9 |
| MVD(Yes/No) | 14/50 | 21.9/78.1 |
| Blood loss (≤300/>300) | 41/23 | 64.1/35.9 |
| Direct Bilirubin (≤4/>4) | 32/32 | 50/50 |
| Total Bilirubin (≤20/>20) | 2/62 | 3.1/96.9 |
| ALT (≤40/>40IU/L) | 39/25 | 60.9/39.1 |
| TNM (I-II/III-IV) | 44/20 | 68.8/31.3 |
| Edmondson Stage (I-II/III-IV) | 58/6 | 90.6/9.4 |
| Tumor location (R/L/R+L) | 18/39/7 | 28.1/60.9/10.9 |
| APRI (≤1/>1) | 46/18 | 71.9/28.1 |
| Neutrophil (≤4/>4) | 40/24 | 62.5/37.5 |
| PT (≤13/>13) | 60/4 | 93.8/6.3 |
| PLT (≤100/>100) | 16/48 | 25/75 |
| AST (≤45/>45) | 39/25 | 60.9/39.1 |
| ALB (≤35/>35) | 3/61 | 4.7/95.3 |
| ICG (≤4.7/>4.7%) | 33/31 | 51.6/48.4 |
| Recurrence (Yes/No) | 33/31 | 51.6/48.4 |
| Liver Cirrhosis (Yes/No) | 63/1 | 98.4/1.6 |

## Supplemental Table 2: Capture probe sequences for EPCAM, CK8/18/19, vimentin, twist, Nanog. and CD45 genes

| **Gene** | **Primers sequences** | |
| --- | --- | --- |
| CD45 | TCGCAATTCTTATGCGACTC | TGTCATGGAGACAGTCATGT |
|  | GTATTTCCAGCTTCAACTTC | CCATCAATATAGCTGGCATT |
|  | TTGTGCAGCAATGTATTTCC | TACTTGAACCATCAGGCATC |
| Twist | ACAATGACATCTAGGTCTCC | CTGGTAGAGGAAGTCGATGT |
|  | CAACTGTTCAGACTTCTATC | CCTCTTGAGAATGCATGCAT |
|  | TTTCAGTGGCTGATTGGCAC | TTACCATGGGTCCTCAATAA |
| Vimentin | GAGCGAGAGTGGCAGAGGAC | CTTTGTCGTTGGTTAGCTGG |
|  | CATATTGCTGACGTACGTCA | GAGCGCCCCTAAGTTTTTAA |
|  | AAGATTGCAGGGTGTTTTCG | GGCCAATAGTGTCTTGGTAG |
| CK19 | CTGTAGGAAGTCATGGCGAG | AAGTCATCTGCAGCCAGACG |
|  | CTGTTCCGTCTCAAACTTGG | TTCTTCTTCAGGTAGGCCAG |
|  | CTCAGCGTACTGATTTCCTC | GTGAACCAGGCTTCAGCATC |
| CK18 | AGAAAGGACAGGACTCAGGC | GAGTGGTGAAGCTCATGCTG |
|  | TCAGGTCCTCGATGATCTTG | CAATCTGCAGAACGATGCGG |
|  | AAGTCATCAGCAGCAAGACG | CTGCAGTCGTGTGATATTGG |
| CK8 | CGTACCTTGTCTATGAAGGA | ACTTGGTCTCCAGCATCTTG |
|  | CCTAAGGTTGTTGATGTAGC | CTGAGGAAGTTGATCTCGTC |
|  | CAGATGTGTCCGAGATCTGG | TGACCTCAGCAATGATGCTG |
| EpCAM | TGGTGCTCGTTGATGAGTCA | AGCCAGCTTTGAGCAAATGA |
|  | AAAGCCCATCATTGTTCTGG | CTCTCATCGCAGTCAGGATC |
|  | TCCTTGTCTGTTCTTCTGAC | CTCAGAGCAGGTTATTTCAG |

## Supplemental Table 3: Comparison of clinical characteristics between two groups of surgical patients

| **Clinical characteristics** | **Laparoscopic** | **Open** | ***P****^#^* |
| --- | --- | --- | --- |
| Operation duration (<242/>242) | 16/16 | 16/16 | 1 |
| blood loss (<300/>300) | 21/11 | 20/12 | 0.794 |
| Pringle Time (<37/>37) | 14/18 | 19/13 | 0.317 |
| Age (≤60/>60 years) | 22/10 | 25/7 | 0.572 |
| Tumor Size (≤5/>5 cm) | 25/7 | 18/14 | 0.062 |
| Differentiation (H/M/L/MIX) | 4/26/2/0 | 5/23/3/1 | 0.739 |
| AFP (<20/20-400/>400 μg/L) | 10/15/7 | 14/9/8 | 0.330 |
| ICG (<4.7/>4.7) % | 13/19 | 20/12 | 0.133 |
| Recurrence（+/-） | 18/14 | 15/17 | 0.453 |
| Metastases（+/-） | 23/9 | 14/18 | **0.042** |

# Laparoscopic hepatectomy group compared with open hepatectomy group

## Supplemental Table 4: The difference in the changes in liver function index before and after operation in the two different operation methods of Lap and Open

| **Variable clinical parameters *** | **Difference between means ± SEM #** | **P value** |
| --- | --- | --- |
| AFP | -7.747 ± 57.63 | 0.8936 |
| AST | -52.84 ± 34.55 | 0.1312 |
| ALT | -55.04 ± 44.13 | 0.217 |
| TB | -1.209 ± 2.528 | 0.6342 |
| WBC | -0.6816 ± 0.8875 | 0.4454 |
| RBC | 0.1972 ± 0.1191 | 0.1029 |
| PLT | 10.06 ± 10.74 | 0.3525 |
| Neutrophilic | -0.8138 ± 0.9178 | 0.3787 |
| APRI | -0.6022 ± 0.3950 | 0.1324 |
| Alb | 0.3125 ± 1.680 | 0.8530 |
| PT | -0.5313 ± 0.6142 | 0.3904 |
| Epithelial CTC | 2.000 ± 0.5701 | **0.0008** |
| Mixed CTC | 11.66 ± 4.918 | **0.0209** |
| Mesenchymal CTC | -0.2813 ± 1.833 | 0.8786 |
| **# Difference between means (OH - LH) ± SEM** | |  |
| *** Variable=Preoperative-Postoperative** | |  |
| **Unpaired t-test** |  |  |

## Supplemental Table 5: Correlation between total CTC and liver function index changes before and after surgery

| **Variable clinical parameters *** | **Increase in total CTC** | |
| --- | --- | --- |
|  | **r** | **P value** |
| AFP | -0.01 | 0.93 |
| AST | -0.22 | 0.08 |
| ALT | -0.20 | 0.12 |
| TB | -0.01 | 0.96 |
| WBC | -0.14 | 0.28 |
| RBC | 0.18 | 0.14 |
| PLT | 0.24 | 0.06 |
| Neutrophilic | -0.15 | 0.23 |
| APRI | -.250* | **0.05** |
| Alb | 0.14 | 0.28 |
| PT | -0.02 | 0.90 |
| Epithelial CTC | **0.45^**^** | 0.00 |
| Mixed CTC | **0.93^**^** | **0.00** |
| Mesenchymal CTC | **0.42^**^** | **0.00** |
| * Variable=Preoperative-Postoperative |  |  |
| Nonparametric correlation |  |  |

# Supplementary Figures

## Supplemental Figure 1


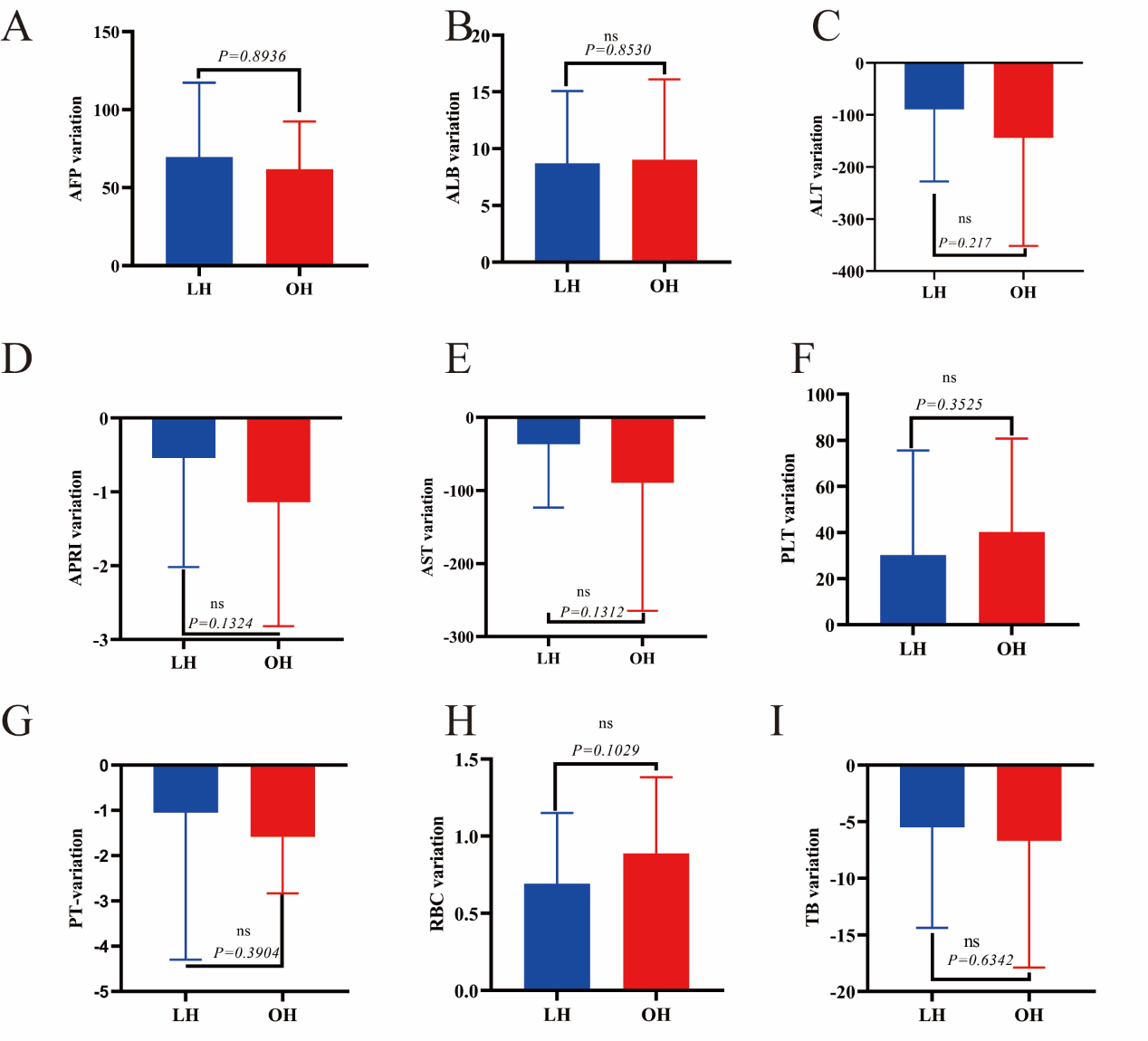


**Supplementary Figure 1.** The difference in the changes in liver function index before and after operation in the two different operation methods of Lap and Open. (A-I) There was no significant difference in the changes in liver function indexes between Lap and Open (P>0.05).

## Supplemental Figure 2


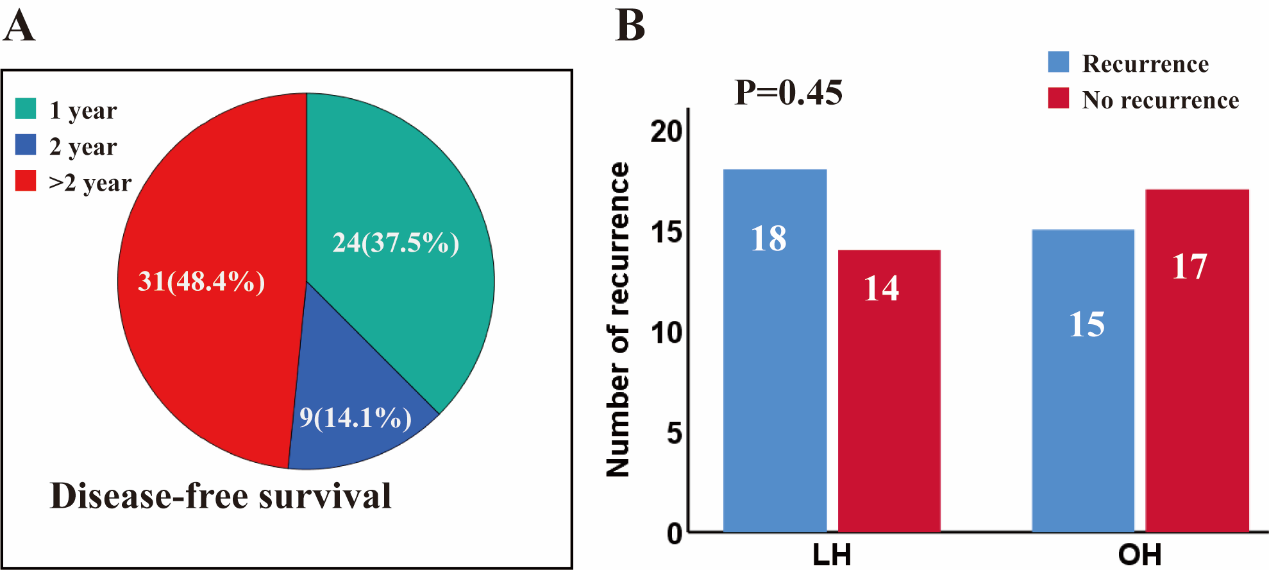


**Supplementary Figure 2. (**A)Statistics of tumor-free survival of 64 patients undergoing liver cancer resection;(B) Classification of surgical methods and recurrence

## Supplementary figure 3


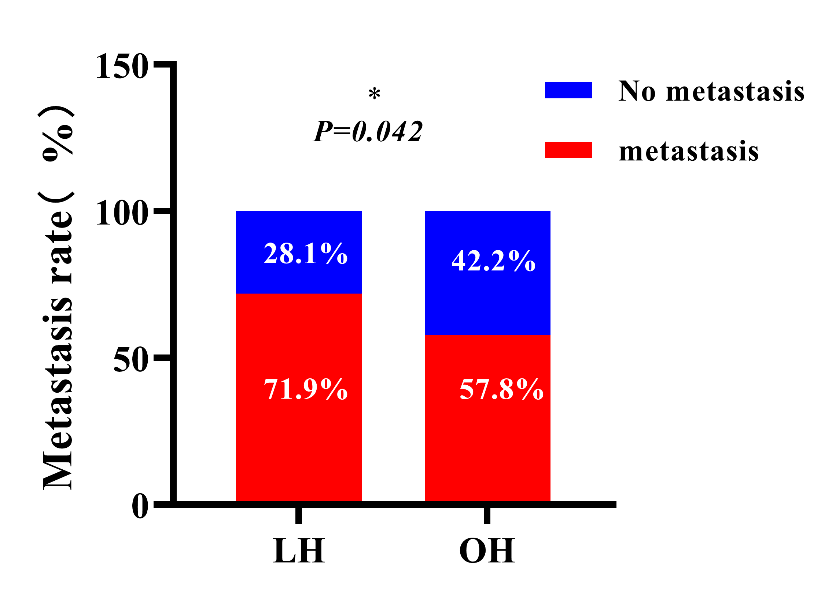


**Supplementary Figure 3.** The intrahepatic metastasis rate in the laparoscopic group was higher than that in the open group (p=0.042).

## Supplementary figure 4


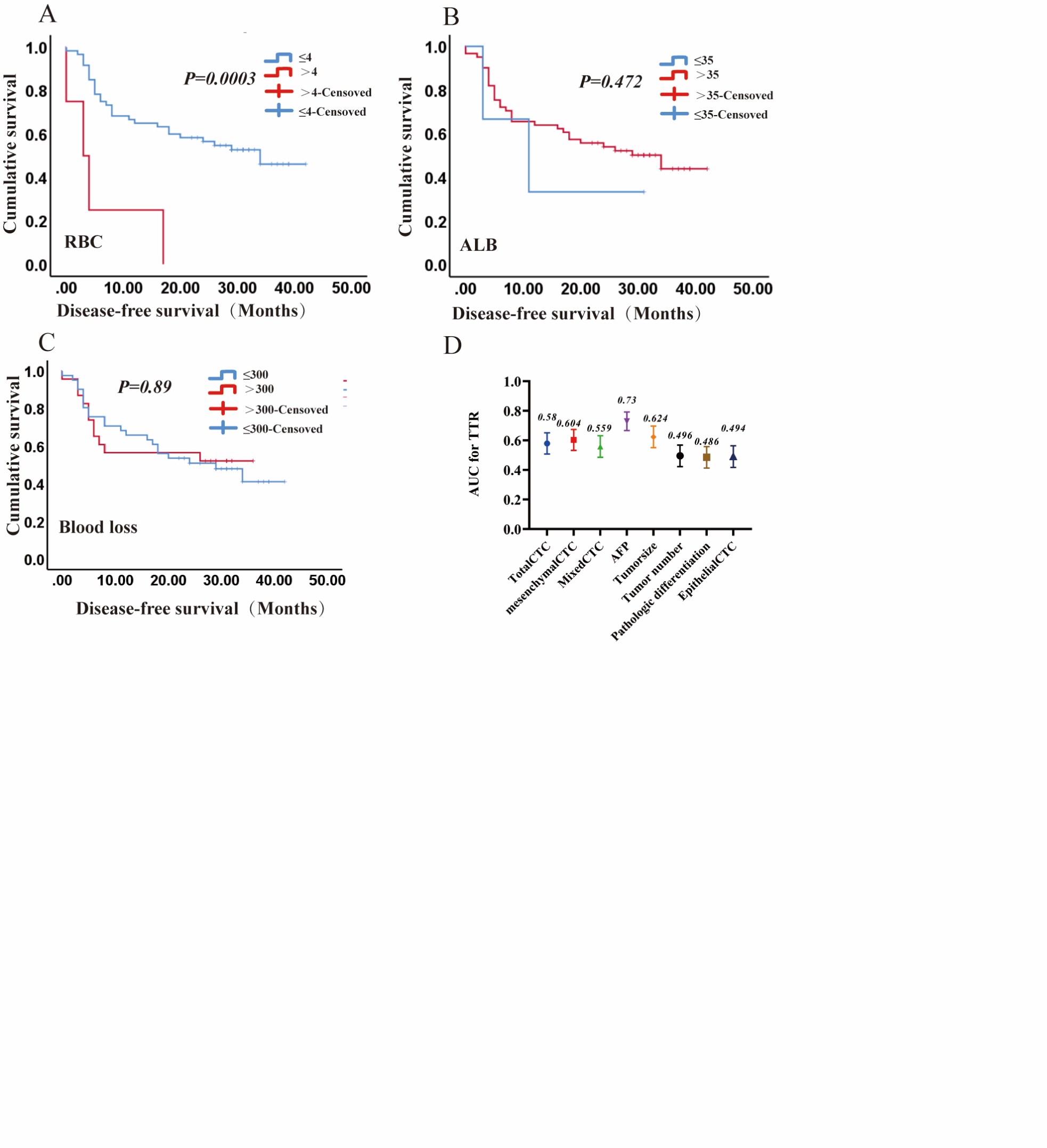


**Supplementary Figure 4.** (A): RBC (red blood cell typing) is associated with recurrence(P<0.05); (B): Albumin typing was not associated with recurrence(P=0.472); (C): Blood loss was not associated with recurrence(P=0.89); (D). ROC curves for total CTC count, Mesenchymal CTC count, Mixed CTC count, Epithelial CTC, AFP, Tumor size, and tumor number.

## Supplementary figure 5


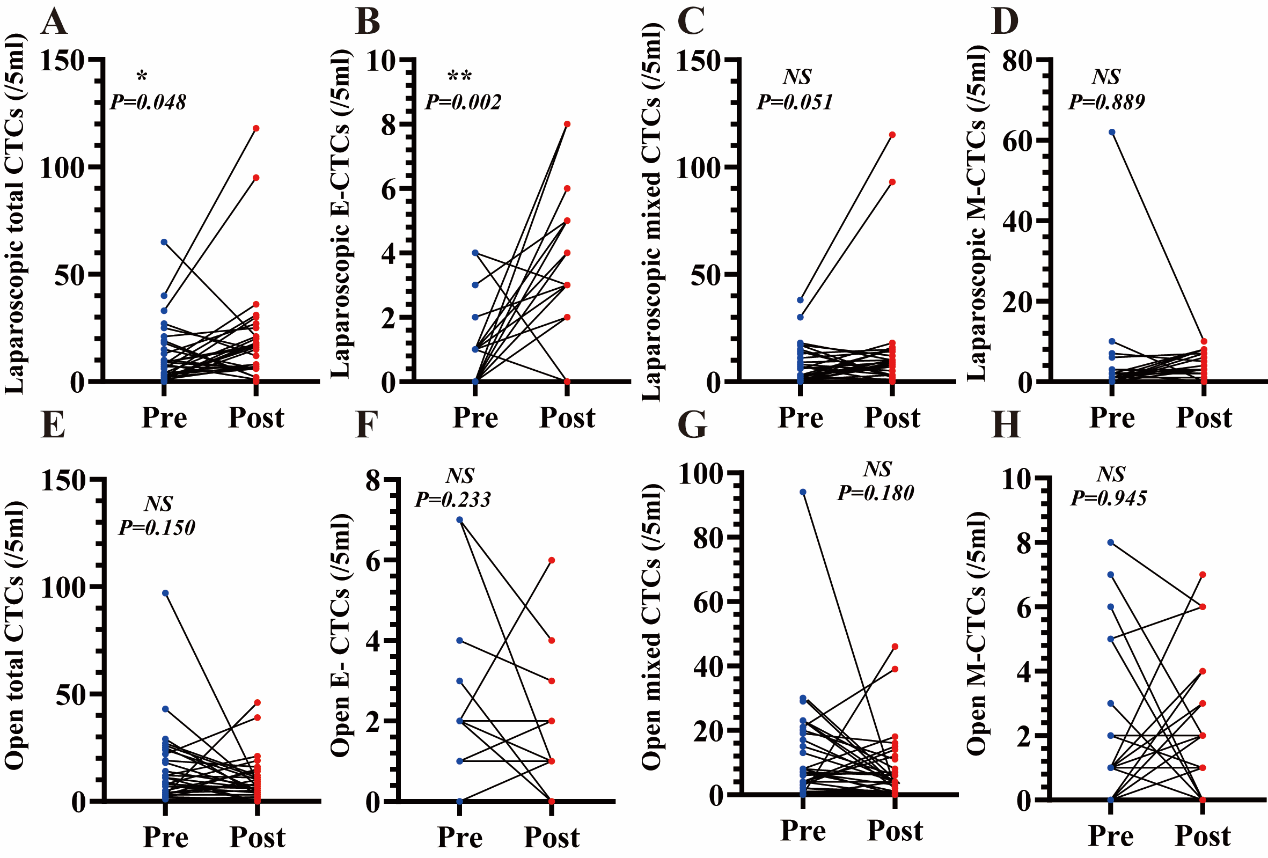


**Supplementary Figure 5.** Changes of total CTCs, epithelial CTCs, mixed CTCs, and interstitial CTCs before and after two types of surgery :(A): Total CTCs increased after laparoscopic hepatectomy (P=0.048); (B): Epithelial CTCs increased after laparoscopic hepatectomy (P=0.002); (C-D) Mixed CTCs and mesenchymal CTCs did not change after laparoscopic hepatectomy (P=0.051,0.889); (E-H) Total CTCs, epithelial CTCs, mixed CTCs, and mesenchymal CTCs did not change significantly after open hepatectomy (P=0.151,0.233,0.180,0.945) ( Paired t-test, graphpad prism).

## Supplementary figure 6


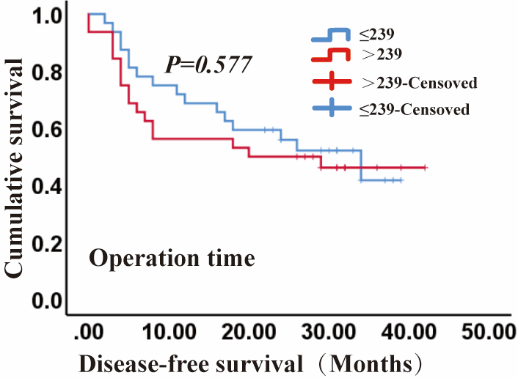


**Supplementary Figure 6.** Operation time was not associated with recurrence(P=0.89)

## Supplementary figure 7


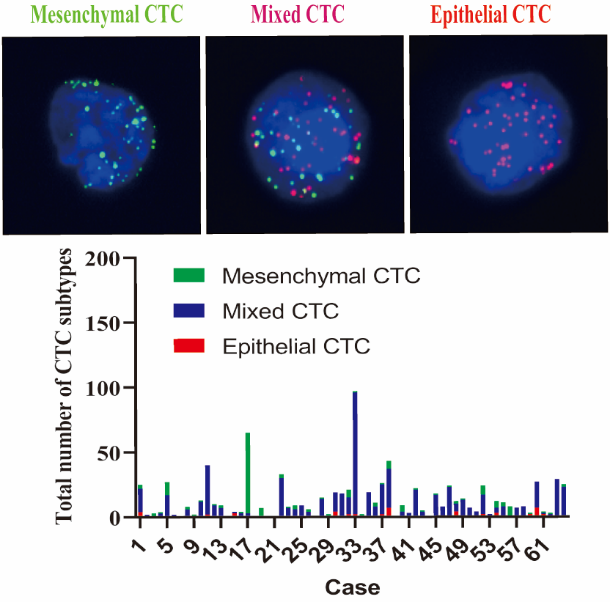


**Supplementary figure 7.** Classification and statistics of CTCs in blood samples from 64 patients with HCC.

## Supplementary figure 8


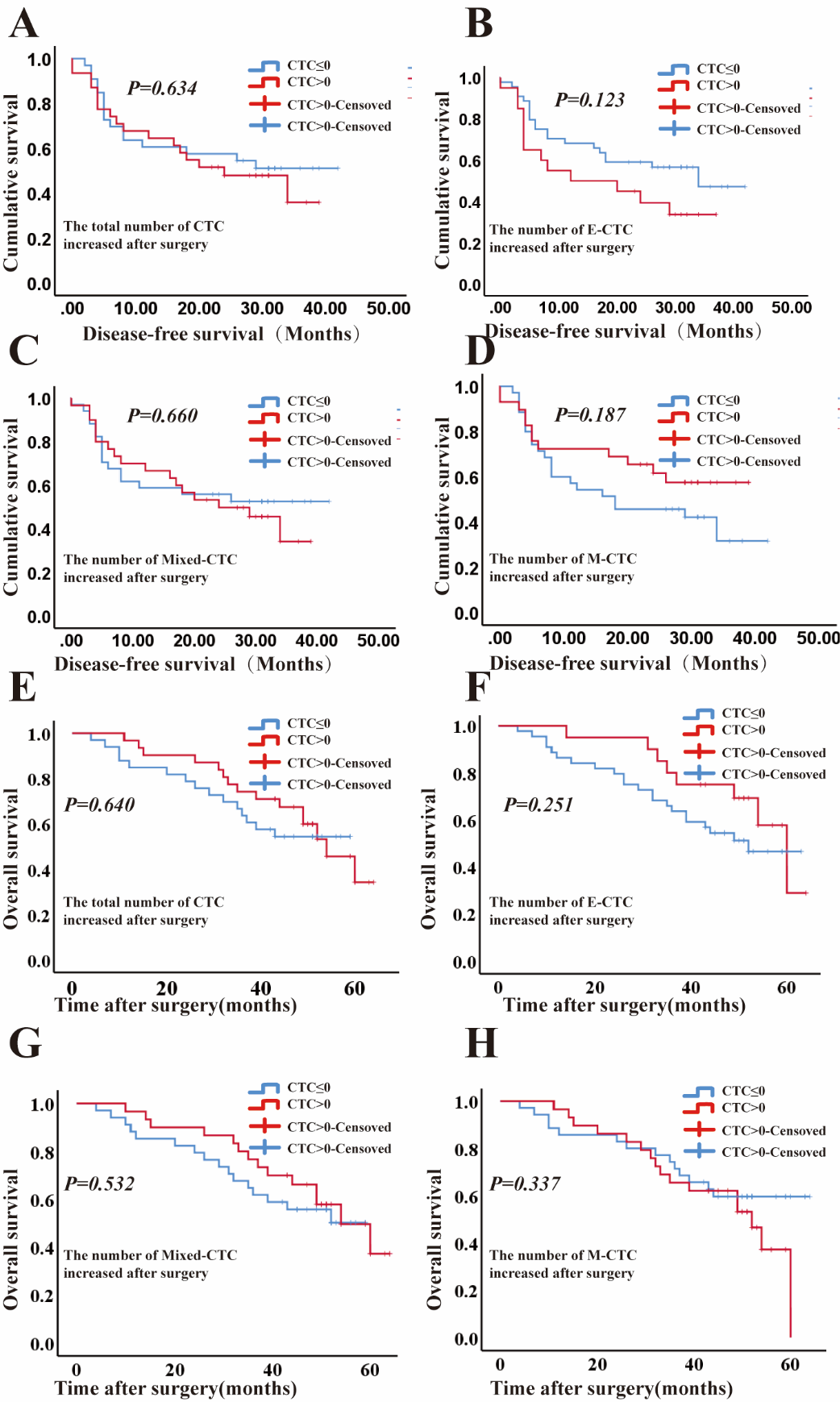


**Supplementary figure 8.** Kaplan-Meier analysis of relapse-free survival and overall survival based on CTCS phenotypes, including increased total, epithelial, mixed and mesenchymal CTCS after surgery. (DFS results by Cox regression analysis)

## Supplementary figure 9

**Supplementary figure 9.** Canpatrol CTCs classification technical procedures：Using a No. 8 blood collection needle and EDTA anticoagulant blood collection tube, collect two tubes (5 ml/tube) of peripheral blood samples. Transfer the pretreated sample into a sample storage tube. Filter the sample, then use a specific capture probe to hybridize with the target mRNA. Subsequently, use an amplification probe to hybridize with the capture probe to prepare for the amplification of the hybridization signal. Label the probe with a fluorophore and hybridize it with the amplification probe to generate a fluorescent signal. Finally, use an automatic identification system to read the fluorescent signal and automatically judge the detection result.
